# Evolution and Future of Glucose Monitoring: From Blood Glucose Meters to Continuous Systems and Their Projected Impact in the Middle East and North Africa (MENA) Region

**DOI:** 10.7759/cureus.100272

**Published:** 2025-12-28

**Authors:** Omar Darkhabani, Abdalla Ahmed

**Affiliations:** 1 Import Management, Nahdi Medical Company, Jeddah, SAU; 2 Medical Microbiology, Noor Biotech, Al Qadarif, SDN

**Keywords:** continuous glucose monitoring, diabetes, flash glucose monitoring, mena region, self-monitoring of blood glucose, technology adoption

## Abstract

The management of diabetes mellitus is fundamentally reliant on the accurate measurement of glycemic levels. For decades, self-monitoring of blood glucose (SMBG) via finger-stick blood glucose meters (BGMs) has been the cornerstone of daily diabetes care. However, the advent of continuous glucose monitoring (CGM) systems represents a paradigm shift, offering real-time interstitial fluid glucose readings, trend data, and hypoglycemic alerts. This review delineates the fundamental principles, developmental history, and comparative advantages and disadvantages of BGM and CGM technologies. We explore the technical evolution from first-generation reflectance meters to modern, connected BGMs and from retrospective professional CGMs to minimally invasive, real-time personal and factory-calibrated systems. Furthermore, we project the future trajectory of these technologies, including non-invasive methods and algorithmic integration. A specific focus is placed on the Middle East and North Africa (MENA) region, which bears one of the world's highest diabetes prevalence rates. We analyze the current market dynamics and project a significant growth in CGM adoption from 2025 to 2035, driven by increasing awareness, competitive pricing, and crucial expansions in healthcare reimbursement, even as BGM remains a vital tool for large segments of the population.

## Introduction and background

Diabetes mellitus is a global pandemic, with the International Diabetes Federation estimating a prevalence of 537 million adults in 2021, a number projected to rise to 643 million by 2030 and 783 million by 2045 [1]. The Middle East and North Africa (MENA) region is disproportionately affected, with some Gulf Cooperation Council (GCC) countries reporting prevalence rates exceeding 20% of the adult population [1,2]. Effective management to maintain glycemic control is critical to preventing devastating micro- and macrovascular complications.

The ability to measure blood glucose is the linchpin of effective diabetes self-management. For over four decades, this was exclusively achieved through self-monitoring of blood glucose (SMBG) using blood glucose meters (BGMs) [3]. While revolutionary in its time, SMBG provides only a snapshot of glucose levels, offering limited insight into glycemic variability, nocturnal hypoglycemia, or post-prandial excursions. The development of continuous glucose monitoring (CGM) systems has addressed these limitations by providing a near-continuous stream of glucose data, trend arrows, and alerts, fundamentally changing the landscape of diabetes care [4].

This review aims to compare and contrast BGM and CGM technologies comprehensively. It will detail their operational principles and technological evolution, systematically evaluate their clinical and practical strengths and weaknesses, and forecast future technological directions. Given the acute public health challenge diabetes poses in the MENA region, a dedicated analysis of the expected adoption and market share dynamics of these technologies in this specific context will be provided.

## Review

Principles and technological evolution

Blood Glucose Meters (BGMs): Principle of Operation

**Figure 1:**
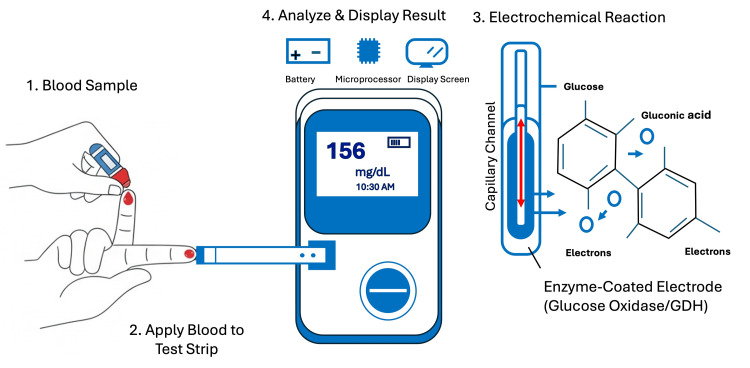
Principle of operation of a blood glucose meter. GDH, glucose dehydrogenase.

The fundamental principle of most modern BGMs is amperometric electrochemical detection [5]. The process involves a disposable test strip containing a capillary channel and an enzyme-coated working electrode. The primary enzymes used are glucose oxidase (GOD) or glucose dehydrogenase (GDH). When a small drop of capillary blood (typically 0.3 to 1 µL) is applied, it is drawn into the strip. The enzyme catalyzes the oxidation of glucose, generating an electrical current that is proportional to the glucose concentration in the blood sample. The meter measures this current, applies an algorithm, and displays the result in units of mg/dL or mmol/L on a digital screen. A simplified schematic of this process is shown in Figure 1.

The development of BGMs can be categorized into distinct generations.

First generation (1970s-80s): The first commercial device, the Ames Reflectance Meter (Dexterity, 1970), was large, expensive, and used reflectance photometry. It required users to time the reaction, wipe the blood off the strip, and then insert it into the meter for a reading [3].

Second generation (1990s-2000s): This era saw the shift to electrochemical strips, enabling "no-wipe" testing. This led to rapid miniaturization, faster results (5-30 seconds), reduced blood volume requirements, and a significant drop in cost, making SMBG accessible to most patients [6,7].

Modern connected generation (2010s-present): Current BGMs focus on connectivity and data management. They feature Bluetooth to sync with smartphone apps for trend analysis, bolus calculators, cloud storage, and easy data sharing with healthcare providers. Alternate-site testing (e.g., forearm) also became available [6]. While alternate-site testing is convenient, the blood from the alternate sites does not always reflect the current glucose level in the central circulatory system (like the blood from the fingertips) [8].

Continuous Glucose Monitors (CGMs): Principle of Operation

CGMs measure glucose concentration in the interstitial fluid (ISF) of the subcutaneous tissue, not in the blood [9]. A small, flexible sensor (containing a glucose-oxidase-coated electrode) is inserted under the skin. As with BGMs, the enzyme reaction generates an electrical signal. This signal is sent wirelessly via a transmitter to a dedicated receiver or a smartphone app. A key physiological consideration is the time lag (typically 5-15 minutes) between blood glucose and ISF glucose levels, particularly during periods of rapid change [10]. Sophisticated algorithms are employed to smooth data, compensate for this lag, and, in many modern systems, calibrate the sensor signal to provide accurate real-time glucose values.

The evolution of CGM has been marked by rapid innovation and can be categorized into distinct generations.

First generation (professional/retrospective): Early systems (e.g., Medtronic MiniMed CGMS, Medtronic, Minneapolis, MN, USA) were used by clinicians to retrospectively download and analyze 72-hour glucose profiles to uncover patterns invisible with SMBG [11].

Real-time CGM (rtCGM): Systems like the Dexcom G4 (Dexcom, San Diego, CA, USA) and Medtronic Guardian introduced real-time glucose display, and customizable alerts and alarms for hypoglycemia and hyperglycemia. These systems typically require periodic calibration with finger-stick BGMs. In addition, the displayed number is accompanied by a directional arrow (trend arrows). These arrows indicate the speed and direction of the glucose change. An arrow pointing straight across (→) means your glucose is relatively stable. An arrow pointing double-up (↑↑) means your glucose is rising rapidly (e.g., 3 mg/dL per minute or more).

Flash glucose monitoring (FGM) and factory-calibrated rtCGM: A major advancement came with Abbott's FreeStyle Libre (FGM) (Abbott, Alameda, CA, USA), which eliminated finger-stick calibration by using a factory-calibrated sensor [12]. The user obtains glucose data and a historical trend graph by scanning the sensor with a reader or smartphone. The latest rtCGM systems (Dexcom G7, Medtronic Guardian 4, FreeStyle Libre 3) are now also factory-calibrated, offering high accuracy, miniaturized designs, longer wear durations (10-14 days), and direct integration with automated insulin delivery systems.

**Figure 2:**
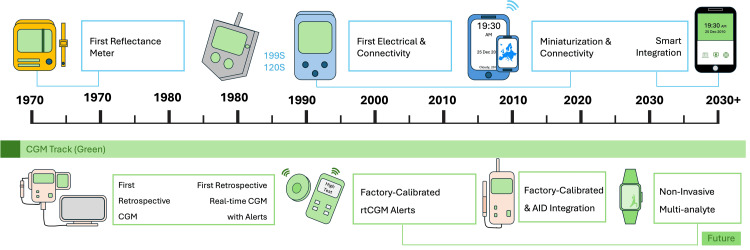
Timeline of development of glucose monitoring device generations. CGM, continuous glucose monitoring; rtCGM: real-time CGM; AID, automated insulin delivery.

A timeline illustrating this concurrent evolution is presented in Figure 2.

Comparative Analysis: Advantages and Disadvantages

A systematic comparison of BGM and CGM technologies is critical for understanding their appropriate clinical application. Table 1 provides a detailed overview.

**Table 1:** Comparative analysis of blood glucose meters (BGM) and continuous glucose monitors (CGM). MARD, mean absolute relative difference.

Feature	Blood glucose meters (BGM)	Continuous glucose monitors (CGM)
Biological sample	Capillary blood	Interstitial fluid
Measurement type	Invasive, intermittent snapshot	Minimally invasive, continuous (e.g., every 1-5 minutes)
Key metrics provided	Single glucose value	Real-time value, trend arrows, historical graphs, time in range (TIR), glucose management indicator (GMI)
Accuracy (MARD)	Typically <5-10%	Modern systems: <10% (Many <8%); MARD improves with each generation (21)
Calibration	Required for every test (via strip)	Factory-calibrated (modern systems) or requires finger-stick calibration (older rtCGM)
Hypo/hyperglycemia alerts	No	Yes, customizable visual and vibratory alarms
Usability & convenience	Requires carrying meter, strips, and lancing device; painful finger-sticks	Sensor worn for 7-14 days; scanning (FGM) or automatic data transmission (rtCGM)
Data for clinical decisions	Essential for calibration and point-in-time insulin dosing	Superior for pattern recognition, adjusting basal insulin, and mealtime dosing with trend data
Cost	Low upfront cost, recurring cost of strips and lancets	High upfront/recurring cost for sensors/transmitters; cost-effectiveness is proven in reducing complications
Primary limitation	No trend data, painful, misses nocturnal events	Physiological lag, sensor insertion, potential skin irritation, higher cost

Future of glucose monitoring and the MENA market: technological directions

The trajectory of glucose monitoring points towards greater integration, intelligence, and minimal invasiveness. The goal is to evolve from a device that simply reports a number into a comprehensive, proactive metabolic health system.

Extended Sensor Longevity: The Quest for Low Maintenance

The current standard of care requires sensor replacement every seven to 14 days, a factor that contributes to cost, inconvenience, and sensor site irritation. Research is heavily focused on developing more stable enzymes and significantly improved biocompatible materials for the sensor components. The aim is to achieve sensors lasting 30 days or longer, which would dramatically reduce the yearly cost of consumables and patient hassle. Longer wear-time also provides a more uninterrupted view of glucose trends, making treatment decisions more informed. This move is essential for boosting patient adherence and the overall cost-effectiveness of CGM technology for healthcare systems [13-15].

Non-invasive and Minimally Invasive Technologies: The Ultimate Goal

The biggest barrier to adherence for people with diabetes remains the need for skin penetration [14-16]. Significant investment is being made in technologies that eliminate or drastically reduce the need for lancing or subdermal insertion. This area is exploring several physics- and chemistry-based approaches.

Optical sensors: Devices using light, such as Raman spectroscopy or near-infrared (NIR) light, attempt to measure glucose concentration based on how light interacts with the molecules in the skin. The main challenge here is overcoming signal interference from other skin components, temperature, and movement.

Transdermal extraction: This involves drawing tiny, painless samples of interstitial fluid through the skin, often using low-level electric currents (reverse iontophoresis).

Biofluids: Research continues into the feasibility of accurately correlating glucose levels in easily accessible biofluids like tears or saliva [17] to the blood glucose concentration. Smart contact lenses are a well-publicized example of tear-based monitoring. While technical challenges remain in ensuring accuracy, consistency, and calibration, a truly non-invasive device is universally recognized as the ultimate technological goal that could revolutionize diabetes screening and management globally.

Multi-Analyte Sensing: The Next Frontier in Metabolic Health

The ability to simultaneously monitor multiple biomarkers offers a more holistic view of metabolic status than glucose alone.

The next generation of implanted or wearable sensors will measure glucose alongside other critical analytes such as ketones and lactate [18]. Simultaneous measurement of blood ketones is vital for patients with type 1 diabetes, providing an early warning system for diabetic ketoacidosis, which is a life-threatening complication. On the other hand, tracking lactate levels is crucial for athletes and can also indicate tissue perfusion issues or severe illness.

Future iterations may include measuring additional biomarkers, such as insulin and C-peptide (to assess insulin production) or cortisol (a stress hormone), allowing for truly comprehensive metabolic tracking and diagnostics beyond diabetes management [19-21].

Market share projection in the MENA region (2025-2035)

The Middle East and North Africa (MENA) region, particularly the Gulf Cooperation Council (GCC) nations, presents a critical and rapidly growing market for diabetes technologies due to a confluence of wealth, high diabetes prevalence, and advanced healthcare infrastructure in many areas.

The current market is dominated by BGM due to its low initial cost, familiarity, and established reimbursement pathways. However, this dynamic is shifting rapidly as awareness and technology accessibility improve. There are several drivers for CGM Growth in the MENA region, including the following. 

High diabetes prevalence: The sheer number of people with diabetes, particularly in countries like Saudi Arabia and the UAE, creates a substantial and concentrated addressable market. The high rates of obesity and type 2 diabetes necessitate effective, scalable management tools [22].

Growing awareness: increased patient and physician education about the clinical benefits of “Time in Range” (the percentage of time a patient spends in the target glucose range, typically 70−180 mg/dL) is the key metric driving demand. Since BGM only provides snapshots, it is insufficient for calculating and improving “Time in Range”, thus accelerating the shift to continuous monitoring [23].

Government initiatives and health economics: as evidence of CGM's superior cost-effectiveness in reducing acute complications (like ketoacidosis) and long-term microvascular and macrovascular complications grows globally, significant pressure will mount on public and private insurers across the MENA region to provide reimbursement. Reimbursement is the single most critical factor for widespread adoption. Government mandates and large-scale public health programs promoting modern diabetes care will unlock mass market potential [22].

Competitive pricing and localization: The entry of multiple domestic and international CGM manufacturers and the eventual introduction of highly competitive, potentially generic sensors will inevitably drive down prices. Furthermore, companies are prioritizing localization by providing Arabic-language marketing, clinical support, and device interfaces, making the technology more accessible and user-friendly to the local population [24,25].

Market share projection: the convergence point

While BGM will remain the most accessible and primary tool for the vast population of non-insulin-treated type 2 diabetics and those in lower-resource settings, the market share dynamics for intensive management are changing fundamentally.

**Figure 3:**
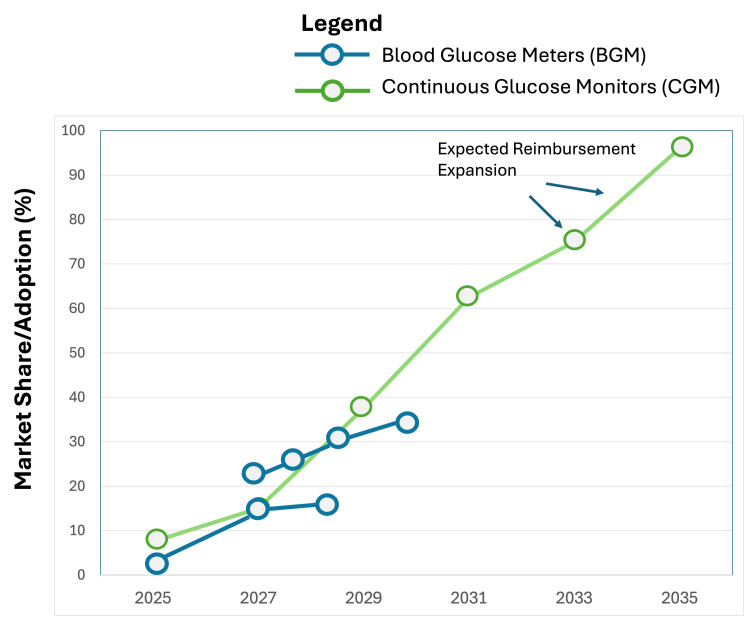
Projected MENA market growth for BGM and CGM (2025–2035), based on current adoption trends and market analysis. MENA, Middle East and North Africa; BGM, blood glucose meter; CGM, continuous glucose monitoring.

CGM is projected to capture a rapidly increasing share of the type 1 and insulin-dependent type 2 diabetes markets. Figure 3 illustrates the projected market growth and the converging shares of BGM and CGM in the MENA region from 2025 to 2035. We anticipate that by 2035, CGM will become the undisputed standard of care for all Type 1 diabetes patients in the GCC and a significant portion of complex Type 2 patients managed with multiple daily injections or insulin pumps. BGM will continue to serve as a vital backup tool (for sensor failure or calibration needs) and remain the primary monitoring tool for budget-conscious and less complex type 2 cases where insulin use is minimal or absent [26,27].

## Conclusions

The journey from the first reflectance meter to modern, connected biosensors exemplifies the profound impact of technology on chronic disease management. Blood glucose meters democratized day-to-day glucose monitoring and will remain an essential, cost-effective tool for millions. However, continuous glucose monitoring systems represent a superior technological paradigm, providing the dynamic data necessary for personalized, proactive diabetes care. For the MENA region, confronting a severe and growing diabetes epidemic, the strategic and accelerated adoption of CGM is not merely a technological upgrade but a public health imperative. Overcoming the barrier of cost through expanded reimbursement will be pivotal in ensuring that these transformative technologies fulfill their potential to improve the quality of life and long-term health outcomes for the millions living with diabetes in the region.
